# A Heat-Stimulated Luminous Fiber Using Heat-Sensitive Green TF-G Pigment

**DOI:** 10.3390/ma11030425

**Published:** 2018-03-15

**Authors:** Yang Jin, Xiaolong An, Mingqiao Ge

**Affiliations:** College of Textile and Clothing, Jiangnan University, 1800 Lihu Avenue, Wuxi 214122, China; ft3103206@163.com (Y.J.); xiaolongan20@163.com (X.A.)

**Keywords:** rare earth, luminescence, thermochromism, luminous fiber, heat sense

## Abstract

In this study, we fabricated a heat-stimulated luminous fiber (HSLF) by wet spinning. The HSLF consists of Sr_2_ZnSi_2_O_7_: Eu^2+^, Dy^3+^ (SZSO), Y_2_O_2_S: Eu^3+^, Mg^2+^, Ti^4+^ (YOS), and heat-sensitive green TF-G pigment (HSGP). SZSO and YOS serve as a source of luminescence to yield a long afterglow system. HSGP is a heat-stimulating agent which develops the link between luminescence and temperature for HSLF. The luminescence of the HSLF is dull below 30 °C, but vivid above 30 °C. The luminescence of HSLFs can be stimulated by low heat (human body temperature). Emission spectra were recorded at 20 °C and 30 °C to investigate the heat-stimulated luminescent performance of HSLFs. HSLF is a smart material which can discern the exciting light to change color because of the photo-thermal effect. This characteristic provides optimum conditions for SZSO and YOS to store energy. The results demonstrated that most luminescence from SZSO and YOS could be absorbed by HSGP at 20 °C, but the luminescence could be liberated at 30 °C. The heat-stimulated phenomenon could also be verified by afterglow and the naked eye.

## 1. Introduction

In recent years, many researchers have focused on luminous fibers, which show practical applications in apparel, wearable devices, safety warning systems, and decorations. The luminescent performance of this functional material is significantly affected by rare-earth luminescent materials, which are blended into the fiber as a source of luminescence. Currently, there are several long-afterglow rare-earth luminescent materials that can be used to prepare luminous fibers, such as Sr_2_ZnSi_2_O_7_: Eu^2+^, Dy^3+^ (SZSO), Sr_2_AlO_3_: Eu^2+^, Dy^3+^ (SAO), Y_2_O_2_S: Eu^3+^, Mg^2+^, and Ti^4+^ (YOS). SZSO, SAO, and YOS present blue, green, and red luminescence emissions, respectively, with long afterglow time and highly efficient energy storage, and have emerged as prominent materials for the preparation of luminous fibers [1,2,3,4]. Luminous fibers prepared from SZSO, SAO, and YOS have diverse luminescence colors owing to the intercoordination of these three rare-earth luminescent materials [5]. A number of luminous fibers with different luminescence colors have been fabricated over the years. For example, a luminous fiber with white light can be fabricated using SZSO, SAO, and YOS by adjusting their ratios [6,7,8,9,10]. A luminous fiber with polychromatic light has many applications in sensors and optical devices [11,12].

**Figure 1:**
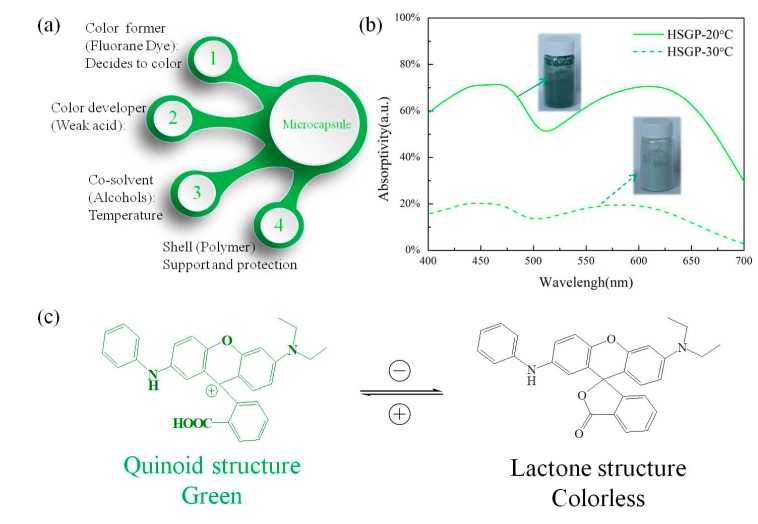
(**a**) Thermochromism of heat-sensitive green TF-G pigment (HSGP); (**b**) The absorption spectra of HSGP at 20 °C and 30 °C; (**c**) Heat-sensitive green TF-G dye with different chemical structures.

Fluorane dye is a widely used heat- or pressure-sensitive leuco dye in security printing, textile coloring, toys, and marketing, because of its rich color and highly sensitive color change property. Three components, namely fluorane dye as the color former, weak acids as the developer, and alcohols as the co-solvent, can be mixed to form a reversible thermochromic system. This reversible thermochromic system is driven by interactions among the three components. The color former is an electron donating compound. The developer is an electron acceptor (proton donor) compound, such as Bisphenol A and phenols [13,14,15]. Typically, the colored complex of a color former and developer prevail below the melting point of the co-solvent. Color change always takes place at the melting temperature of the co-solvent. When the co-solvent melts, the dye–developer complex is destroyed, and the system acquires the natural color of fluorane dye. Such a reversible thermochromic system can be encapsulated in a polymer envelope to form a microcapsule as a pigment [16,17,18,19,20]. This microcapsul enables this solid–liquid thermochromic system to retain the solid powder, which can be directly blended with a polymer to fabricate fiber or film materials for further applications. The structure of the microcapsule is shown in Figure 1a.

In this study, we used heat-sensitive green TF-G pigment (HSGP) to fabricate a heat-stimulated luminous fiber. The absorption spectra of the HSGP are shown in Figure 1b. The inset images show the HSGP at different temperatures. The absorption spectra of the HSGP at 20 °C show two absorption peaks at 460 nm and 610 nm. These two absorption bands cover the blue band (400–500 nm) and the red band (500–700 nm), respectively. According to the structural colorations, these two strong absorption peaks arise from the quinoid conjugate structure of heat-sensitive green TF-G dye. As shown in Figure 1c, heat-sensitive green TF-G dye donates electrons and transforms into a quinoid structure. The conjugation degree of the lactone structure is lower than that of the quinoid structure; when heat-sensitive green TF-G dye transforms into the lactone structure at 30 °C, the absorption of the colorless lactone structure is pushed into the ultraviolet region. Hence, heat-sensitive green TF-G dye becomes colorless. The remaining absorbance in the visible band is caused by the residual heat-sensitive green TF-G dye, which results from an incomplete transformation in the microcapsule. Because of their characteristic of color change, we use SZSO and YOS as the luminous sources to generate blue and red luminescence to complement the color change property of HSGP. HSGP was utilized as a heat-stimulating agent that develops the link between heat and luminescence at low temperature [21]. The theoretical thermochromic temperature of HSGP is 28 °C (this can be verified by differential scanning calorimetry). The thermostability of HSGP is insufficient for melt spinning, but wet spinning is a suitable method to prepare heat-stimulated luminous fibers [22,23,24]. Therefore, polyacrylonitrile (PAN) is the best carrier to obtain the fiber material because of its good spinnability. Moreover, PAN has outstanding photostability and can serve as the matrix of the luminous fiber [25].

## 2. Experimental

### 2.1. Raw Materials

SrCO_3_, ZnO, SiO_2_, Y_2_O_3_, S, TiO_2_, 4MgCO_3_·Mg(OH)_2_·6H_2_O, Eu_2_O_3_, Dy_2_O_3_, H_3_BO_3_, Na_2_CO_3_, and dimethyl sulfoxide (DMSO) of analytical reagent grade were purchased from Sinopharm Chemical Reagent Co., Ltd., (Shanghai, China). Polyacrylonitrile (PAN) powders were produced from Shaoxing Gimel Advanced Materials Technology Co., Ltd. (Shaoxing, China). Reversible heat sensitive green TF-G pigment (HSGP) was purchased from Shenzhen Aobo security anti-counterfeiting technology Co., Ltd. (Shenzhen, China).

### 2.2. Preparation of Rare-Earth Luminescent Materials

Sr_1.95_ZnSi_2_O_7_: Eu^2+^_0.02_, Dy^3+^_0.03_ and Y_2_O_2_S: Eu^3+^_0.04_, Mg^2+^_0.05_, Ti^4+^_0.05_ were prepared using a high-temperature solid-state method. After preliminary milling, the raw materials were dissolved in alcohol followed by mechanical mixing for 15 min and ultrasonic dispersion for 30 min to obtain the homogeneous mixture. Sr_1.95_ZnSi_2_O_7_: Eu^2+^_0.02_, Dy^3+^_0.03_ were heated with 8 mol. % H_3_BO_3_ at 1400 °C for 3 h in a reducing atmosphere; the Y_2_O_2_S: Eu^3+^_0.04_, Mg^2+^_0.05_, Ti^4+^_0.05_ was synthesized by adding 15 mol. % Na_2_CO_3_ as the flux at 1300 °C for 3 h in a reducing atmosphere. The sintered products were milled in a ball mill and sieved to obtain the desired size.

### 2.3. Fabrication of the Heat-Stimulated Luminous Fiber

Rare-earth luminescent materials and HSGP were added to homogeneous PAN/DMSO spinning dope as the spinning solution. The ratio of PAN/DMSO was 20% in terms of mass-volume concentration. The mass ratios of HSGP are 3%, 5%, and 8% to the PAN/DMSO spinning dope. Samples with 0 wt %, 3 wt %, 5 wt %, and 8 wt % HSGP are respectively labeled as HSLF0, HSLF3, HSLF5, and HSLF8. Sr_1.95_ZnSi_2_O_7_: Eu^2+^_0.02_, Dy^3+^_0.03_ and Y_2_O_2_S: Eu^3+^_0.04_, Mg^2+^_0.05_, and Ti^4+^_0.05_ were uniformly mixed with the PAN/DMSO spinning dope in mass ratios of 4% and 6%. Wet spinning was carried out using a syringe (needle type: 22 G) and a boost device. The prepared spinning solution was injected into water at room temperature (20–25 °C) with an injection speed of 4 mL/min to obtain the heat-stimulated luminous fiber (HSLF).

### 2.4. Characterization

The microstructure of the rare-earth luminescent materials and HSLFs was examined by scanning electron microscopy (SEM, Japan Hitachi TM3030, Chiyoda, Tokyo, Japan). The particle size was computed from the SEM images using Nano Measurer 1.25 software. X-ray diffraction (XRD) patterns were recorded using a Bruker D8 Advance (Germany) at room temperature; the samples were scanned from 10° to 70° at 4°/min using a Cu anode. Differential scanning calorimetry (DSC) measurements were performed using a Q200 manufactured by TA Instruments (New Castle, DE, USA); the temperature was varied from 0 °C to 350 °C at 10 °C/min. Emission spectra were recorded at 20 °C and 30 °C using a FS5 fluorescence spectrometer manufactured by Edinburgh Instruments (Livingston, UK), for which the slit was 2 nm in width, excitation wavelengths ranged from 400 to 700 nm, and the dwell time was 0.2 s; the samples were excited by 365 nm ultraviolet (UV) light for 5 min before tests. Absorption spectra were acquired using a Macbeth Color-Eye 7000A (Grand Rapids, MN, USA) color measuring and matching instrument. Afterglow decay curves were obtained using a PR-305 afterglow brightness meter by exciting the samples at 1000 lx for 5 min. All images were taken using a Canon G15 camera; the images in the bright field were recorded under an artificial daylight 6500K (D65) illumination source. Before all optical tests, samples were kept in darkness for 20 h to rule out error.

## 3. Result and Discussion

### 3.1. Microstructure

**Figure 2:**
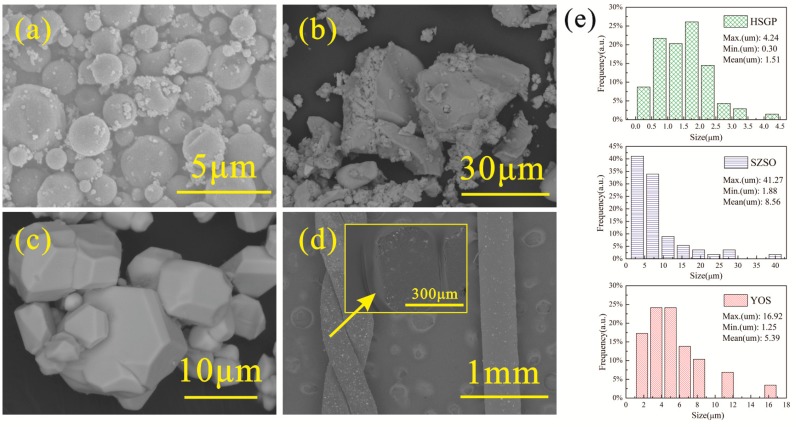
SEM images of (**a**) HSGP; (**b**) SZSO; (**c**) YOS; and (**d**) HSLF5. The inset image in (**d**) is the cross section of HSLF5; (**e**) Particle size distributions of HSGP, YOS, and SZSO.

Figure 2a shows that the microstructure of HSGP is spherical. SZSO and YOS were synthesized by a high-temperature solid-state method and were then milled to a powder (Figure 2b,c, respectively). Particle size distributions of HSGP, SZSO, and YOS are shown in the bar chart (Figure 2e). The particle sizes of HSGP, SZSO, and YOS were 0.3–4.24 µm, 1.88–41.27 µm, and 1.25–16.92 µm, respectively. The prepared spinning solution was extruded from the syringe needle. The HSLFs were drawn into water by gravity. The microstructure of HSLF5 is shown in Figure 2d. The HSLFs are uniform and cylindrical in shape. The diameter of the cylinder ranges from 410 to 420 mm owing to the inner diameter of the needle (410 mm). Deformation took place due to twisting. The cross section (inset image in (d)) of the HSLFs appears elliptical. The HSLFs can be twisted by tension, indicating that they have sufficient strength for further fabrication. Clearly, many particles are exposed on the fiber surface, whereas some are buried in the PAN matrix.

### 3.2. X-Ray Diffraction Analysis

**Figure 3:**
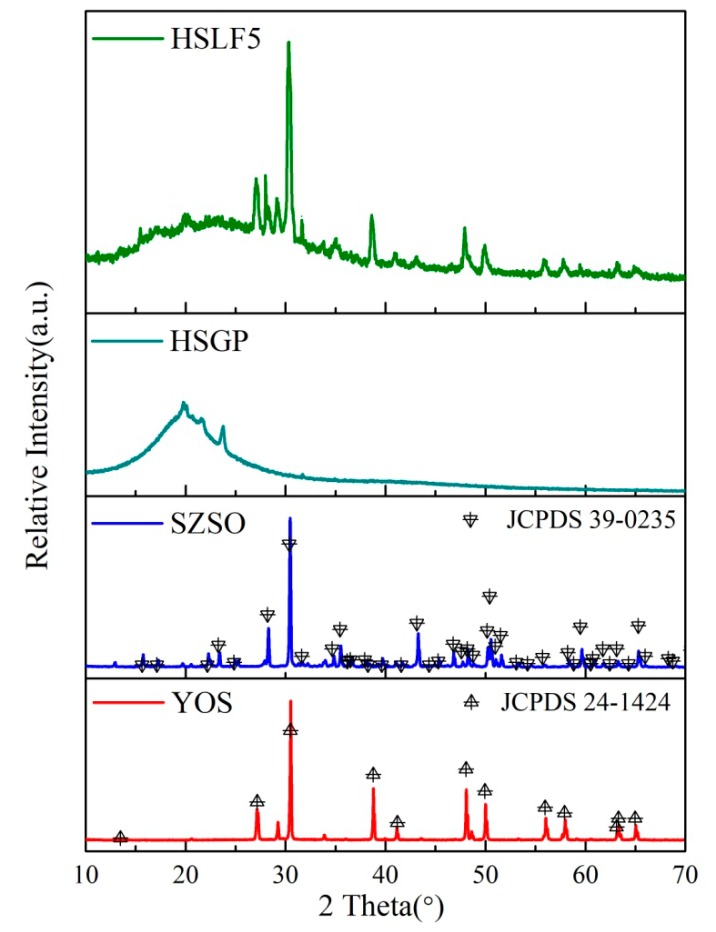
XRD patterns of HSLF5, HSGP, and rare-earth luminescent materials.

Figure 3 shows the XRD patterns of HSLF5, HSGP, SZSO, and YOS. The diffraction peaks of SZSO and YOS can be indexed well to a cubic cell with tetragonal and hexagonal crystal systems with space groups P-421m and P-3m1; this is in good agreement with data from the standard Joint Committee on Powder Diffraction Standards Cards (JCPDS) (No. 39-0235 and No. 24-1424). This suggests that rare-earth ions occupy the chemical substitutional sites of Sr_2_ZnSi_2_O_7_ and Y_2_O_2_S, but this occupancy does not disturb the crystal structure. The characteristic peaks of HSGP and rare-earth luminescent materials can be found in the XRD pattern of HSLF5, indicating that the crystal phases of the rare-earth luminescent materials and HSGP were not destroyed during milling and spinning.

### 3.3. Differential Scanning Calorimetry Analysis

**Figure 4:**
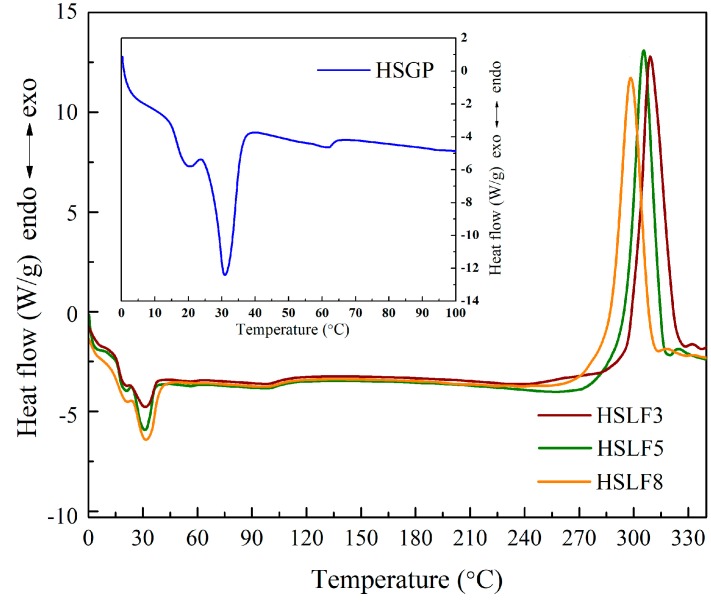
DSC curves of HSLFs and HSGP (inset).

DSC curves of HSLFs and HSGP are shown in Figure 4. Two peaks can be seen on the curves of the HSLFs. The endothermic peak close to 30 °C corresponds to the DSC curve of HSGP. This peak arises from the melting of the co-solvent, which decides the thermochromic temperature in the HSGP reaction system. The endothermic peak intensity is directly related to the content of HSGP with increasing ratio of the co-solvent. The curve for the thermochromic pigment shows that the co-solvent begins melting at 20 °C and is completely melted at 30 °C. The melting temperature of the co-solvent matches the thermochromic temperature. The exothermic peak near 300 °C is caused by the decomposition of PAN, because cyanide decomposes at a temperature close to the melting temperature of PAN. Moreover, the triple bond of cyanide in PAN will release a large amount of heat when ruptured. Therefore, the heat released by the breakage of chemical bonds manifests as an exothermic peak. The integral values of HSLF3, HSLF5, and HSLF8 are 115.25, 76.38, and 75.32, respectively. The integrals of the exothermic peaks gradually reduce with an increase of HSGP, as the blended particles influence the molecular arrangement of PAN when the fiber is formed and drafted in the coagulating bath. This causes PAN to release less heat when it decomposes.

### 3.4. Absorptivity Analysis

**Figure 5:**
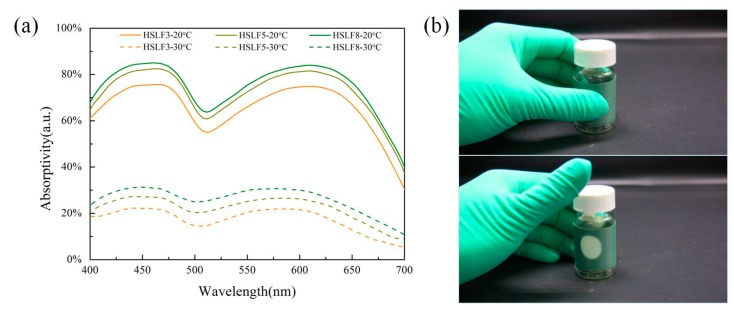
(**a**) Absorption spectra of HSLFs at 20 °C and 30 °C; (**b**) Thermochromicity of HSLF5 induced at body temperature.

The absorption spectra of HSLFs in Figure 5a demonstrate the absorption ability of HSLFs in different temperature conditions. The color change properties of HSLFs are affected by the HSGP in the fiber. The absorption spectra of HSLFs exhibit similar waveforms to those of HSGP. The difference between HSGP and HSLFs is their absorptivity. The absorptivity of HSLFs slightly exceeds that of HSGP. Moreover, the absorptivity of HSLF is measured in terms of ratios of HSGP; thus, it increases with the content of HSGP. HSLF5 was entwined on a glass flask to demonstrate thermochromism. Figure 5b shows images demonstrating the thermochromism of HSLF5 induced by body temperature under artificial daylight 6500K (D65). The chromaticity coordinate of HSLF5 is (0.2898, 0.3761) at room temperature (20 °C). On touching the sample with a finger, the region touched by the finger changes in color to white, and the chromaticity coordinate is (0.3144, 0.3381). This indicates that the heat required to drive HSLFs to change color is very low. Hence, HSLFs are novel thermochromic fibers that can be used to sense heat flow from the human body.

### 3.5. Emission Spectra Analysis

**Figure 6:**
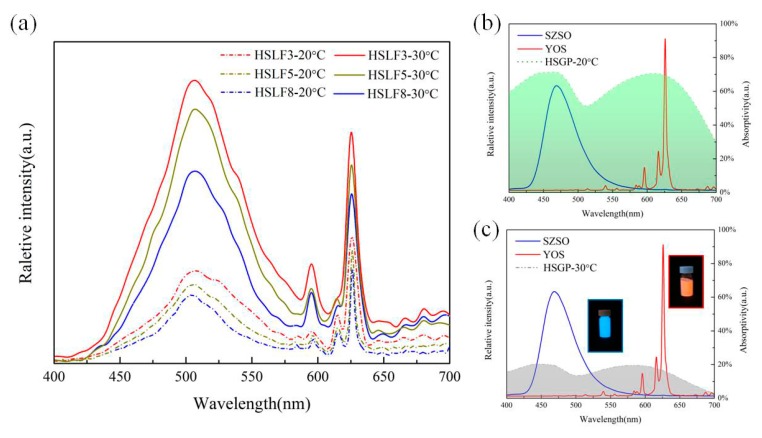
(**a**) Emission spectra of HSLFs at 20 °C and 30 °C. Emission spectra of YOS and SZSO, and the absorption spectra of HSGP, at (**b**) 20 °C and (**c**) 30 °C.

Figure 6a shows the emission spectra of the HSLFs. Figure 6b,c show the emission spectra of SZSO and YOS, and the absorption spectrum of HSGP. The emission spectra and absorption spectra were acquired at 20 °C and 30 °C. From the emission spectra of SZSO and YOS, it is evident that the emission peaks of SZSO at 470 nm are generated by the representative 4f^6^ to 5d^1^ transition from Eu^2+^. The emission peak of trivalent dysprosium is not observed, because Dy^3+^ does not act as a luminescence center in SZSO. However, Dy^3+^ is the co-doping ion which generates the hole trap to improve afterglow. The red emission of YOS is comprised of several significant emission peaks generated from the ^5^D_0_ → ^7^F_J_ (J = 0, 1, 3, 4), ^5^D_1_ and ^5^D_2_ → ^7^F_J_ (J = 0, 1, 2, 3, 4) transitions of Eu^3+^ and the ^5^D_0_ → ^7^F_2_ transition of Eu^3+^. Bivalent magnesium and tetravalent titanium act as assistive ions, which significantly promote the afterglow of YOS. In Figure 6b,c, the emission spectra of SZSO and YOS are compared with the absorption spectrum of HSGP. HSGP shows strong absorptivity in the blue and red regions at 20 °C. This strong absorption suggests that the luminescence of SAO and YOS cannot be transmitted through HSGP. The strongest absorption of HSGP at 470 and 610 nm is higher than 70%, which overlaps with the emission of SZSO and YOS. In this case, most of the energy will be absorbed by HSGP in the fiber. This indicates that HSGP has a filtering capacity, and can completely shield the SZSO and YOS luminescence. As shown in Figure 6a, the emission spectra of the HSLFs show several emission peaks. The emission peak at 500 nm arises from SZSO, and the characteristic emission peaks at 596, 616, and 626 nm arise from YOS. The emission peak of SZSO in the blue band located at 470 nm is very close to the absorption peak of HSGP (460 nm), and the emission peaks of HSLFs are located at 505 nm, which is exactly the location of the trough (500 nm). The peak near 470 nm is covered by HSGP, and the energy near the trough is easy to transmit; therefore, the emission peak of SZSO red-shifts from 470 nm to 505 nm owing to the influence of HSGP on the spectrum. Although varying the content of HSGP does not change the emission peaks of HSLFs, the luminescence intensities of the various HSLFs are significantly different. The integral of the emission spectra, which illustrates the luminescence intensity in the visible regions, is shown in Table 1; it demonstrates the effect of temperature on intensity. Intensity is related to the temperature and content of HSGP. For the same temperature, intensity is inversely proportional to the content of HSGP. The effect of temperature is distinct. It can be seen that the luminescence from SZSO and YOS is almost shielded by HSGP at 20 °C. When the temperature is 30 °C, the emission intensity is much stronger than at 20 °C. The decreased absorption of HSGP in the visible band leads to high transmittance of the luminescence from SZSO and YOS. The increment in luminescence intensity is almost 200%. The luminescence of SZSO and YOS can easily transmit through HSGP possessing a lactone structure; this observation is consistent with the spectra of HSLFs obtained at 30 °C. Therefore, HSLFs have a heat-stimulated luminescence capacity which can be induced by low heat flow.

### 3.6. Heat-Stimulated Luminescence Property

**Figure 7:**
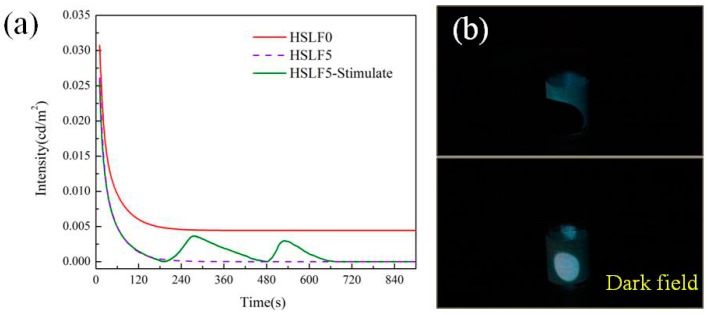
(**a**) Afterglow decay curve of HSLF5 and HSLF0. HSLF5, which is stimulated by heat flow, is labeled as HSLF5-Stimulate; (**b**) Heat-stimulated luminescence phenomenon of HSLF5 stimulated by the touch of a finger.

The afterglow decay curve of HSLF5 is shown in Figure 7a. The afterglow decay curve of HSLF0 has also been included to demonstrate the effect of HSGP on afterglow. The decay curves were calculated by fitting the decay data using the following equation:

$$I\;=\;A_{0}\;+\;A_{1}\cdot \exp\left(-\frac{t}{\lambda_{1}}\right)\;+\;A_{2}\cdot \exp\left(-\frac{t}{\lambda_{2}}\right)\;+\;A_{3}\cdot \exp\left(-\frac{t}{\lambda_{3}}\right)$$

where I is the luminescence intensity; A_0_ is the initial value; t is the time; A1, A2, and A3 are constants; and λ_1_, λ_2_, and λ_3_ are the decay times for the exponential components. The values of these parameters for the samples are listed in Table 2. The numerical relationship between λ_1_, λ_2_, and λ_3_ indirectly suggests the decay processes of HSLF0 and HSLF5. λ_1_, λ_2_, and λ_3_ respectively reveal an initial rapid decay, subsequent medium decay, and final stable decay. When t is 0, the initial luminescence intensity can be calculated. The initial luminescence intensity of HSLF5 and HSLF0 is 0.03026 cd/m^2^ and 0.03328 cd/m^2^, respectively; these values are similar. Thus, it can be deduced that the absorption efficiencies of HSLF5 and HSLF0 are very similar because HSGP does not shield much of the exciting light. This manifests in the excellent color change ability of HSLF, which can be induced by low heat. The co-solvent is melted by the exciting light because of the photo-thermal effect (see Supplementary Materials). Driven by the liquated co-solvent, the HSGP exhibits low absorptivity in the visible band. This improves the absorption capacity and reserve energy of SZSO and YOS. This novel property makes HSLFs a smart functional fiber, which can discern exciting light to activate the absorption model. After removing the exciting light, the color of the HSLF changes to green and is accompanied by a reduction in temperature. From the afterglow decay curve, the decay speed of HSLF5 is much faster than that of HSLF0 in the initial stage because the luminescence was shielded by HSGP. This indicates that HSLF perceives the disappearance of excited light and turns on the shield model. We used heat flow to stimulate HSLF5 at 180 s for 120 s. A salient peak can be seen on the afterglow curve of HSLF5-Stimulate, and the peak value is close to that of HSLF0. After stopping the heat flow, the afterglow intensity gradually declines with time. To verify that this heat-stimulated property is reproducible, we turned on the heat flow again at 480 s. The luminescence intensity enhances again. Figure 7b shows the HSLF5 stimulated by heat from a finger. The position of contact shows distinct luminescence, whereas this luminescence is dimmer at other positions. The luminescence caused by touch is clearly visible. HSLF thus has potential applications in smart devices, functional clothing, and heat sensors.

## 4. Conclusions

In this study, we fabricated HSLF, which consists of HSGP, SZSO, YOS, and PAN. The fibers are columnar with diameters of 410 µm. XRD results demonstrated that milling did not destroy the crystal structure of the rare-earth luminescent materials, which were prepared using a high-temperature solid-state method. Moreover, the crystal structures of SZSO, YOS, and HSGP were not disturbed by spinning; hence, the properties of the rare-earth luminescent materials and HSGP were retained in the fiber. DSC results showed that the co-solvent completely melted at 30 °C, indicating that heat-sensitive green TF-G dye transforms from a quinoid structure to the lactone structure above 30 °C. Absorptivity analysis indicates that there are two absorption peaks of HSLFs, which are close to the emission peaks of SZSO and YOS. HSLFs turn white in color at 30 °C, and this thermochromism can be induced the human body temperature. Emission spectra demonstrate that the absorption of HSGP overlaps the emission of SZSO and YOS; thus, HSGP can absorb luminescent energy from SZSO and YOS at 20 °C. In contrast, the luminescence intensity of HSLFs is enhanced by approximately 200% at 30 °C. HSLF is a smart material that can discern excitation light and open light patches for energy storage. After removing the excited light, HSLFs can shield the luminescence of the inner fiber. Heat-stimulated luminescence can be induced by low heat flow: even heat from the human finger can drive the heat-stimulated luminescence of HSLFs. The luminescence is brilliant and vivid, thus exhibiting potential applications in smart garments, heat sensors, and inductors.

## Figures and Tables

**Table 1:** Integral area of emission spectra.

Sample	Integral Area		Ratio(30 °C/20 °C)	Increment
	20 °C	30 °C		
HSLF3	90,716.70	246,481.86	2.72	171.71%
HSLF5	67,544.30	204,112.11	3.02	202.19%
HSLF8	52,807.11	161,821.93	3.06	206.44%

**Table 2:** Fitting parameters of afterglow curves.

Sample	A_0_	A_1_	A_2_	A_3_	λ_1_	λ_2_	λ_3_	R^2^
HSLF0	0.00443	0.01276	0.00248	0.01361	10.25226	2.25055	51.60385	0.9996
HSLF5	0.0000103	0.00225	0.01304	0.01496	8.66898	1.45401	46.29428	0.99881

## Supplementary Materials

The following are available online at http://www.mdpi.com/1996-1944/11/3/425/s1.
