# Supplementary material for: A Heat-Stimulated Luminous Fiber Using Heat-Sensitive Green TF-G Pigment
Source: Materials (Basel). 2018 Mar 15;11(3):425. doi: 10.3390/ma11030425 (PMC5873004; doi:10.3390/ma11030425)
Supplement: Supplementary file 1 [file materials-11-00425-s001.pdf]

Materials **2017**, *7*, 425; doi:10.3390/ma11030425

Supplementary Materials: A Heat-Stimulated Luminous Fiber Using Heat-Sensitive Green TF-G Pigment

Yang Jin, Xiaolong An, and Mingqiao Ge *

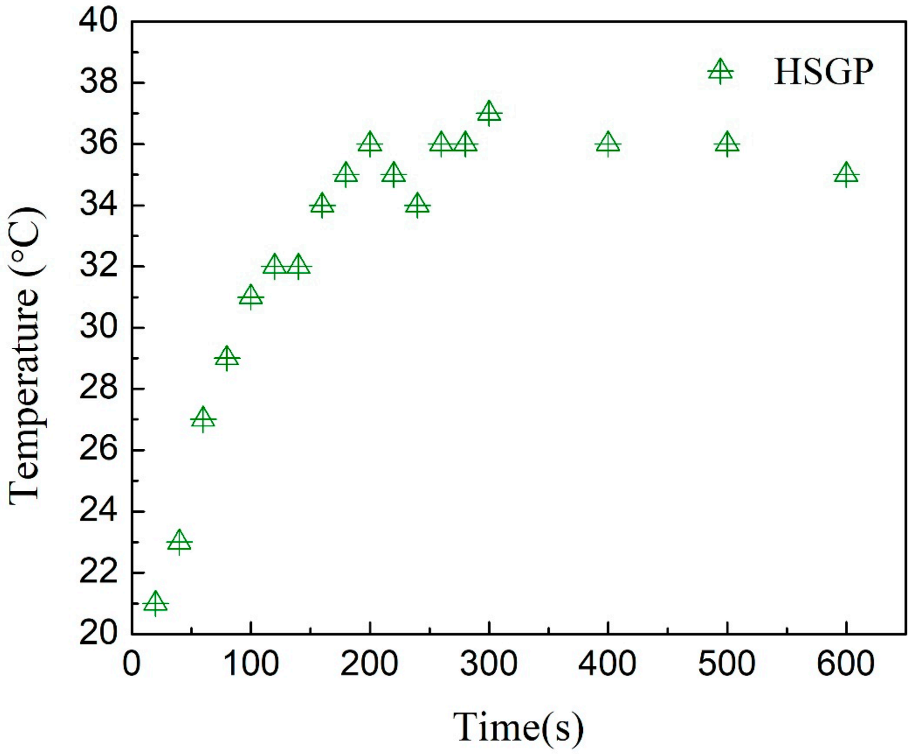

Figure S. 1g HSGP was placed under 1000lx light source and recorded the temperature of HSGP (room temperature is 20°C).
